# Novel caged luciferin derivatives can prolong bioluminescence imaging *in vitro* and *in vivo*†

**DOI:** 10.1039/c8ra02312c

**Published:** 2018-05-30

**Authors:** Chaochao Zhang, Mingliang Yuan, Guangxi Han, Yuqi Gao, Chunchao Tang, Xiang Li, Lupei Du, Minyong Li

**Affiliations:** Department of Medicinal Chemistry, Key Laboratory of Chemical Biology (MOE), School of Pharmacy, Shandong University Jinan Shandong 250012 China mli@sdu.edu.cn +86-531-8838-2076 +86-531-8838-2076; State Key Laboratory of Microbial Technology, Shandong University Jinan Shandong 250100 China; Shenzhen Research Institute, Shandong University Shenzhen Guangdong 518057 China

## Abstract

Based on *N*-cyclobutylaminoluciferin (cybLuc), a set of high and efficient caged bioluminescent derivatives (Clucs) as firefly luciferase pro-substrates has been developed herein. After careful examination, these molecules exhibited low cytotoxicity and prolonged bioluminescence imaging up to 6 h *in vitro* and *in vivo*. Importantly, these caged luciferin derivatives have the potential to serve as long-term tracking tools to explore some biological process by using bioluminescent imaging.

## Abstract 2

A set of high and efficient caged luciferin derivatives exhibited low cytotoxicity and prolonged bioluminescence *in vitro* and *in vivo*.

## Graphical abstract

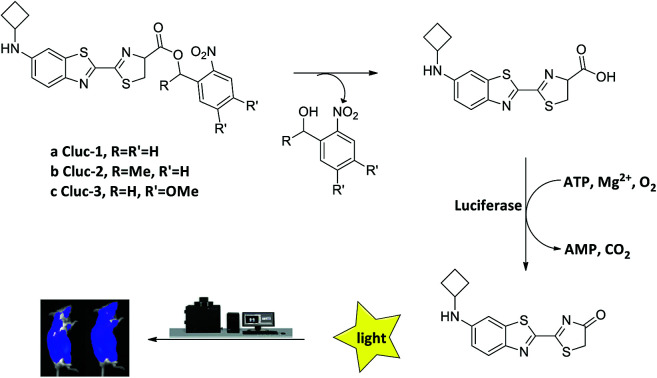

Bioluminescent imaging (BLI) is a consistently sensitive, convenient, reliable and non-invasive imaging technique that has been comprehensively applied for monitoring a myriad of life processes,^1^ including cell proliferation and migration,^2^ tumor growth,^3^ protein–protein interactions,^4^ enzyme activities^5^ and other uses.^6^ The firefly luciferase-luciferin system is one of the most commonly used among the approximate 30 different bioluminescence systems.^7^ In the last two decades, to expand the application of firefly luciferase-luciferin bioluminescent imaging *in vitro* and *in vivo*, a lot of effort has been made.^8^ A mass of caged luciferin analogues has been well developed, which could serve as highly responsive bioluminescent probes for specific biomolecules.^9^ For instance, in order to test various enzymes activity, several bioluminescent probes have been well designed for aminopeptidase N,^10^ nitroreductase,^11^ β-galactosidase,^12^ as well as H_2_O_2_.^13^ Meanwhile, modified luciferins have also been developed, such as an alkyne-modified luciferin,^14^ 6-substituted luciferin analogues,^15^ luciferin analogues containing a six-membered oxazine ring^16^ and a selenium analogue of firefly d-luciferin.^17^ However, the broad application of the luciferin is limited to strong attenuation of bioluminescent signals that are emitted below 600 nm, which results from the absorption and scattering of light by tissue.^18^ Moreover, the relatively short circulatory half-life of luciferin *in vivo* is another important factor to consider.

Recently, caged compounds, shielded by protecting groups have been utilized to distantly control the cellular functions^19^ or to investigate unknown biological phenomena.^20^ These the bio-inactive caged compounds can generate the biological activity when the protecting groups are removed.^21^ Specifically, a novel genetic technique for photo-mediated temporal and spatial control of gene activation in zebrafish embryos has been demonstrated as an alternative to the gene knockdown approach by using antisense, morpholino-modified oligonucleotides.^22^ In line with this direction, several caged luciferins have been well developed. For example, four bisdeoxycoelenterazine derivatives were synthesized which displayed the potential for improving the bioluminescence resonance energy transfer assays with reasonable light signal sustainability.^23^ Moreover, ten novel pro-substrates for Renilla luciferase were designed and synthesized by introducing ester protecting groups into the carbonyl group of the free luciferin, which could be efficiently activated by intracellular esterases or other hydrolases leading to a bioluminescence enhancement.^24^ As a result, caging strategy is very promising to prolong the time of bioluminescent imaging.

Recently, we introduced *N*-cyclobutylaminoluciferin (cybLuc) with robust red-shifted light emission and long-term emission which were higher than those of d-luciferin and aminoluciferin.^25^ In the current study, we designed and synthesized a series of caged compounds (Clucs, a–c, Scheme 1) based on cybLuc to prolong the bioluminescence duration. These caged compounds are proposed to release the free luciferins in an aqueous solution, which subsequently interact with firefly luciferase to generate the bioluminescent signals. It should be noted that among all three compounds, the caged luciferin 2 (Cluc-2) displayed prolonged bioluminescent imaging *in vivo* so that it may serve as a promising pro-substrate for BLI in diagnostic and therapeutic fields.

**Scheme 1:**
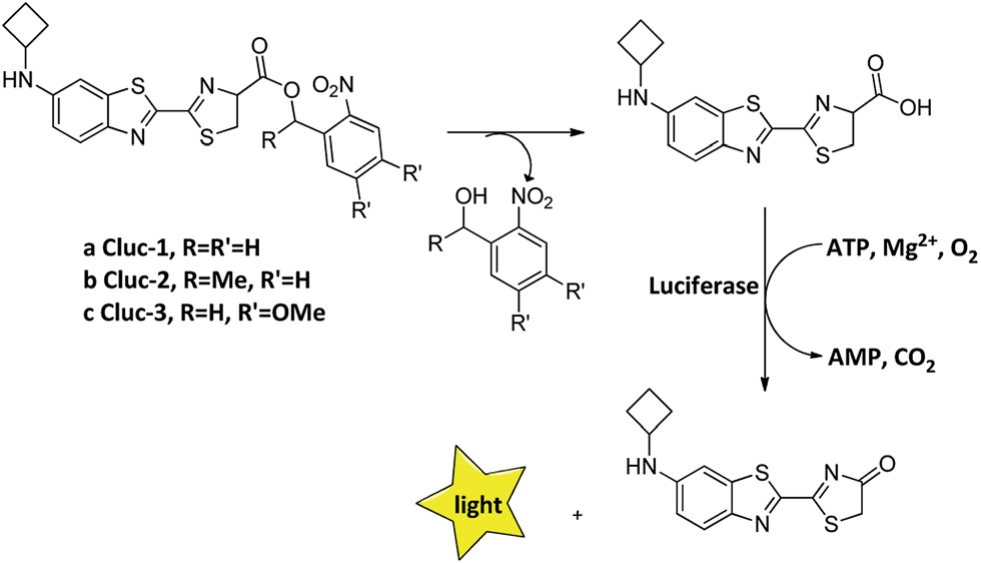
The structure of caged luciferin substrates that can release the substrates of firefly luciferase.

Our caged luciferin derivatives can be conveniently prepared with a facile and efficient method^26^ as depicted in Scheme S1.† In brief, 1-(2-nitrophenyl)ethanones reacted with hydrazine hydrate in dry ethanol under reflux to provide 2-nitrobenzylidene-hydrazines, which were oxidized by activated manganese(iv)-oxide to obtain 1-(diazomethyl)-2-nitrobenzenes. A subsequent reaction of 1-(diazomethyl)-2-nitrobenzenes with cybLuc generated the caged derivatives (a–c). The details for the preparation of the compounds and their ^1^NMR, ^13^C NMR and ESI-HRMS spectra can be found in the ESI.†

After obtaining the products, to test whether these compounds can release cybLuc in the aqueous solution, these caged luciferin derivatives at various concentrations (0–40 μM) were examined in Tris–HCl buffer (100 μL). The equal volume of Tris–HCl buffer containing 20 μg mL^−1^ luciferase and 2 mM ATP were added, and bioluminescence signals were detected with an exposure time of 1 s. As shown in Fig. 1, light emission intensities were enhanced with increasing caged luciferin derivatives concentration. These caged compounds displayed similar dose-dependent manner, and the Cluc-1 had the highest bioluminescence intensity. Overall, these interesting results indicated that these caged luciferin derivatives could release luciferins in Tris–HCl buffer so that they could serve as luciferin pro-substrates using in bioluminescence imaging.

**Fig. 1:**
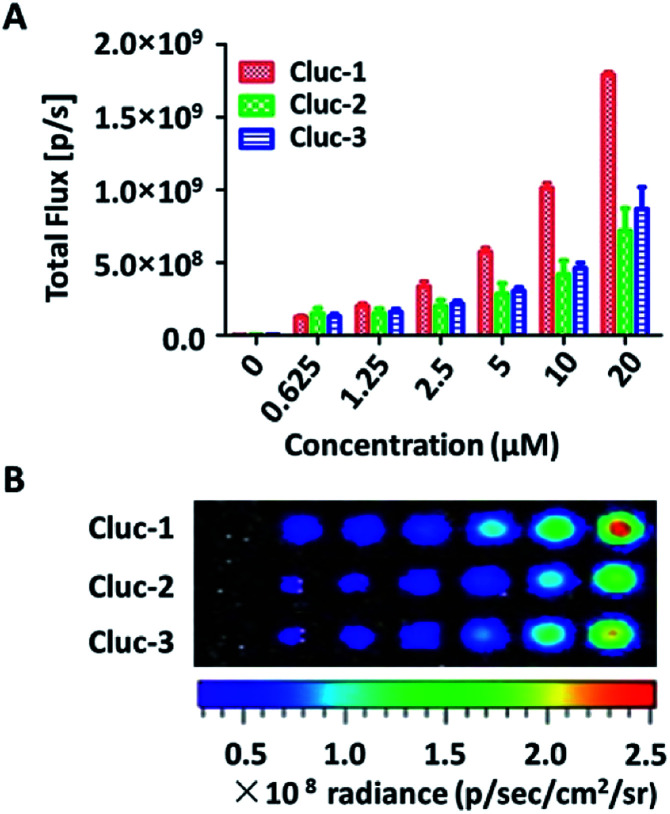
(A) The comparison of bioluminescence intensities of caged luciferin derivatives at different concentrations with firefly luciferase. (B) Bioluminescence imaging of caged luciferin derivatives with firefly luciferase.

The caged luciferin derivatives as imaging agents should be low cytotoxicity. Therefore, we used the way of Cell Counting Kit-8 to investigate the cell viability. The ES-2-Fluc cells (8 × 10^3^ cells per well) were incubated in 96-well plates and the culture medium was removed after 24 h. Then a variety of dilutions of the caged luciferin derivatives in complete growth medium were added. After 12 h incubation, the solution of CCK-8 was added. Finally, the absorbance signals were recorded by a microplate reader at 450 nm, and no marked cytotoxicity was observed (Fig. S1†). The IC_50_ values of these caged luciferin derivatives were much greater than the concentration (40 μM) used in cell imaging, demonstrating good biocompatibility with live cells. Accordingly, we could further investigate the properties of caged luciferin derivatives.

To evaluate the properties of caged luciferin derivatives in BLI at the cellular level, bioluminescence signals from ES-2-Fluc cancer cell lines with constitutive expression of Fluc were analysed. ES-2-Fluc cells were passed and plated (4 × 10^4^ cells per well) in 96-well plates and cultured 24 h. Then the medium was removed and increasing concentrations of caged luciferin derivatives in Tris–HCl buffer (1.25 μM, 2.5 μM, 5 μM, 10 μM, 20 μM and 40 μM) were added. Meanwhile, ES-2-Fluc at various (0, 1250, 2500, 5000, 10 000, 20 000 and 40 000 cells per well) were passed and plated in 96-well plates and incubated for 24 h. Subsequently, the caged luciferin derivatives (40 μM) were added after removing the medium. The bioluminescence was measured at once with an exposure of 10 s and the bioluminescence intensity was collected. As shown in Fig. 2, with increasing concentrations of caged luciferin derivatives, the bioluminescent intensities increased in a dose-dependent manner. Similarly, the bioluminescent intensities of caged luciferin derivatives increased with increasing amounts of cells (Fig. 3A). Overall, these three different caged luciferin derivatives exhibited analogical properties that could release the substrates and produce similar bioluminescence intensity in cell. These results demonstrated that these caged luciferin derivatives could be potent luciferin bioluminescence substrates in the cellular level.

**Fig. 2:**
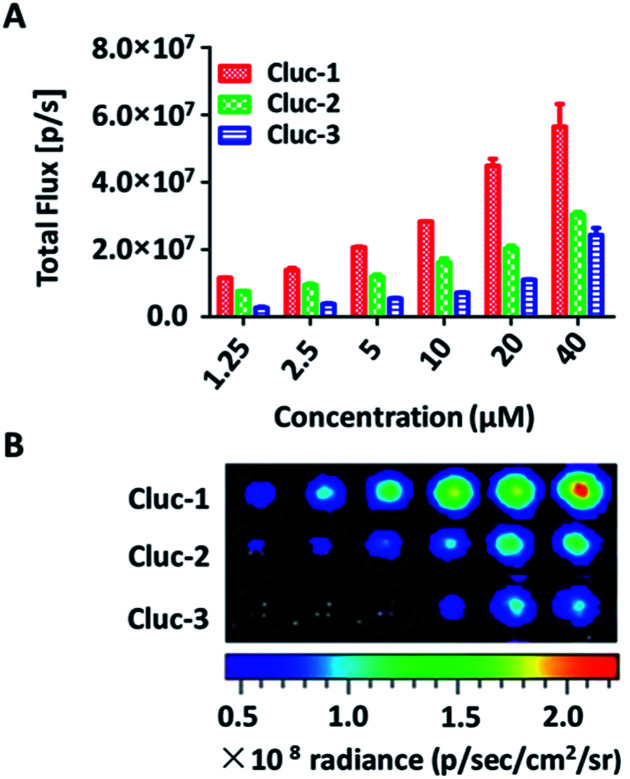
(A) Bioluminescence imaging of caged luciferin derivatives in ES-2-Fluc with different concentrations. (B) Bioluminescence imaging of caged luciferin derivatives in ES-2-Fluc.

**Fig. 3:**
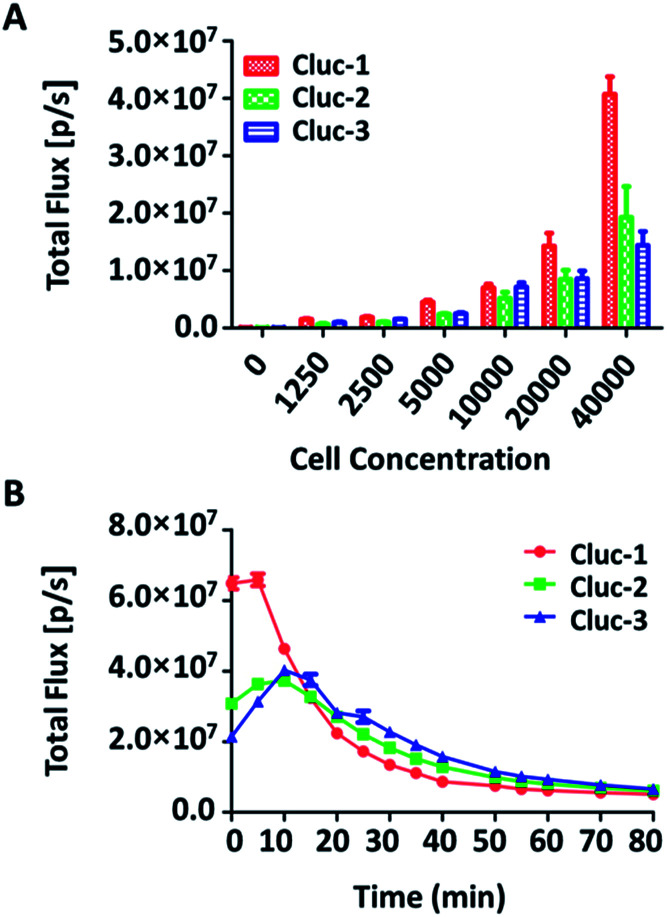
(A) Bioluminescence imaging of substrates incubated with various concentrations of ES-2-Fluc cells and quantification of the bioluminescent imaging signals. (B) The rate of the bioluminescence reaction for the caged luciferin derivatives evaluated as the change of the total light intensity in the cell over time.

To further explore whether the caged luciferin derivatives can serve as a long-term tracking tool, a real-time imaging experiment was conducted at the cellular level. The bioluminescence signal was recorded every five minutes after ES-2-Fluc cells were treated with the caged luciferin derivatives. In brief, cybLuc displayed rapid bioluminescent signal decay and lost approximately 90% of its initial light intensity at 10 min (Fig. S2†). In contrast, other caged luciferin derivatives especially Cluc-2 and Cluc-3 exhibited a longer time to peak and slower kinetics characteristics (Fig. 3B). Moreover, the bioluminescent intensities of compounds Cluc-2 and Cluc-3 could stay about 1 h until they lost most of its light intensity even if they were lower than that of cybLuc. In general, these caged luciferin derivatives could release cybLuc slowly and prolong the time of bioluminescence imaging in cell. These promising results prompted us to employ the caged luciferin derivatives for bioluminescence imaging in living animals.

The potential of caged luciferin derivatives for the bio-imaging *in vivo* was further explored. Pathogen-free luciferase-expressing transgenic mice (about 24 g, female) were provided. CybLuc and caged luciferin derivatives were injected intraperitoneally (100 μL of 1 mM solutions in NS) into mice after they were anesthetized. Then bioluminescence imaging was immediately acquired with an exposure time of 1 s and the light signal was recorded every ten min until it disappeared. Every compound was evaluated using at least three mice. As depicted in Fig. 4, the bioluminescent intensity gradually increased to a maximum and then decayed over time. There existed differences between cybLuc and caged luciferin derivatives about the time of the maximum peak. In the case of cybLuc, the bioluminescence signal peaked at 20 min after injection then declined at a fast rate. However, the signals of Cluc-1 and Cluc-3 reached a plateau after 90 min and then dropped rapidly. And the bioluminescent intensity appeared at 40 min following Cluc-2 injection into the mice. Importantly, the light signal of Cluc-2 could be detected after 6 h. However, the bioluminescent intensities of these caged luciferin derivatives were also reduced as the same as the cellular level. These results indicated that the caged luciferin derivatives especially Cluc-2 were hydrolysed at a slower rate and they were more suitable for stable and long-time imaging study. Moreover, even if these three caged luciferin derivatives exhibited analogical bioluminescence intensity and dose–response analysis *in vitro*, the vivo imaging profile of Cluc-2 was different from Cluc-1 and Cluc-3. We propose that the presence of benzylic methyl group may affect the cell penetration or increase the steric hindrance and decrease its hydrolysis rate, and so that compound Cluc-2 could prolong the time of bioluminescence imaging *in vivo*. Therefore, these caged luciferin derivatives, particularly Cluc-2, could be used for exploring its application in biological fields.

**Fig. 4:**
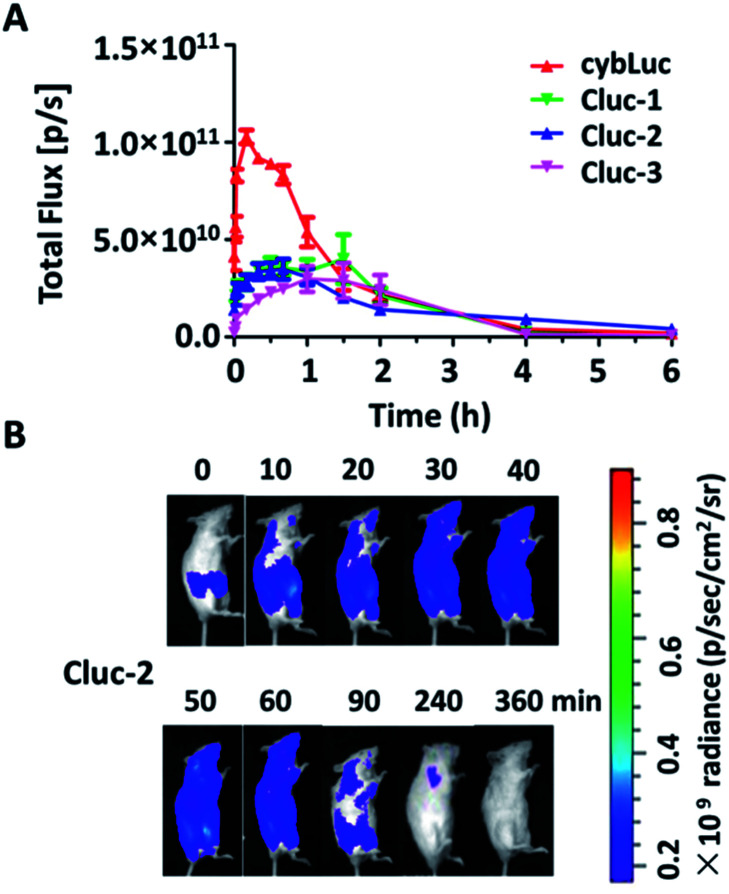
(A) The rate of the bioluminescent reaction for the caged luciferin derivatives and cybLuc evaluated as the change of the total light intensity *in vivo* over time. (B) Bioluminescence imaging for the caged luciferin 2 *in vivo* over time.

## Conclusions

In conclusion, herein we designed and synthesized a series of caged luciferin derivatives based on cybLuc and investigated their bioluminescence properties with firefly luciferase, and further displayed their applications both *in vitro* and *in vivo*. The experimental results clearly demonstrated that these caged luciferin derivatives can easily release the luciferin in Tris–HCl buffer and they displayed decent performance in the bioluminescence assay, so as to be practical pro-substrates of luciferase. Moreover, not only did they indicate a reasonable positive correlation between the concentration and bioluminescent intensities, but also, they implied a longer time and slower kinetics in cell. After careful evaluation *in vivo*, we found that they hydrolysed at a slower rate and got the luminous peak at prolong time. Surprisingly, the light signal of the caged luciferin 2 (Cluc-2) is suitable for stable and long-time imaging study since its signal can be detected after 6 h administration. Compared with our previously luciferin derivative cybLuc, our newly caged derivatives had longer duration at the same dose for *in vivo* bioluminescence imaging. As a whole, these caged luciferin derivatives have the potential to serve as novel luciferase pro-substrates to explore some biological process in relevant fields by using bioluminescent imaging.

## Conflicts of interest

There are no conflicts to declare.

## Supplementary Material

RA-008-C8RA02312C-s001
