# Supplementary material for: Novel caged luciferin derivatives can prolong bioluminescence imaging in vitro and in vivo
Source: RSC Adv. 2018 May 30;8(35):19596–9. doi: 10.1039/c8ra02312c (PMC9080648; doi:10.1039/c8ra02312c)
Supplement: RA-008-C8RA02312C-s001 [file RA-008-C8RA02312C-s001.pdf]

Electronic Supplementary Material (ESI) for RSC Advances.
This journal is © The Royal Society of Chemistry 2018

Supporting Information for

Novel caged luciferin derivatives can prolong bioluminescence imaging *in vitro* and *in vivo*.

Chaochao Zhang,[a] Mingliang Yuan,[a] Guangxi Han,[a] Yuqi Gao,[a] Chunchao Tang,[a] Xiang Li,[a] Lupei Du[a] and Minyong Li[a,b,c*]

[a] Department of Medicinal Chemistry, Key Laboratory of Chemical Biology, School of Pharmacy, Shandong University, Jinan, Shandong 250012, China.

[b] State Key Laboratory of Microbial Technology, Shandong University, Jinan, Shandong 250100, China

[c] Shenzhen Research Institute, Shandong University, Shenzhen, Guangdong 518057, China

Materials

All chemicals were purchased from commercial suppliers and used without further purification. The buffer solution used in the bioluminescent measurement was the 50 mM Tris-HCl buffer (pH 7.4) containing 10 mM $MgCl_2$. Ultrapure water was purified with a Mill-Q filtration system. Pathogen-free luciferase-expressing transgenic mice (FVBTg(CAG-luc,-GFP)L2G85Chco/FathJ17) were obtained from the Jackson Laboratory. All animal studies were approved by the Ethics Committee and IACUC of QiLu Health Science Centre, Shandong University and were conducted in compliance with European guidelines for the care and use of laboratory animals.

Instruments

NMR spectral data were obtained for ^1H NMR and ^{13}C NMR at 400 MHz and 101 MHz, respectively. Chemical shifts are reported in ppm from TMS as the internal reference with the solvent resonance in DMSO-d_6 solution. ESI-HRMS spectral data were obtained by using a Water SYNAPTG2 mass spectrometer. High pressure liquid chromatography (HPLC) spectra and the purity of the compounds were determined by analytical reverse-phase HPLC (Agilent, 1200 Infinity) on a Phenomenex C-18 column (250 × 4.6 mm). The pH test was performed using a pH-meter (OHAUS, STARTER3100). Melting points were measured using a Mel-Temp apparatus and were not corrected. Luminescence imaging was recorded using an IVIS Kinetic (Caliper Life Sciences, USA) equipped with a cooled charge-coupled device (CCD) camera. Circular specified regions of interest (ROIs) were drawn over the areas, and the total flux was calculated using the Living Image software version 4.0 (Caliper Life Sciences).

Synthesis and structural characterization of caged luciferin derivatives.

2-nitrobenzylidenehydrazine (1a)

In a three-necked flask, 2-nitrobenzaldehyde (400 mg) was heated to 80 ^oC and hydrazine hydrate (265 μL, 5.3 mmol) was added slowly. The mixture was stirred at this temperature for 2 h in dry ethyl alcohol. After cooled, the organic phase was evaporated under vacuum. Then pure water was added and the mixture was extracted with DCM (15 mL × 3). The combined organic layer was washed with brine, dried over Na_2SO_4 and concentrated. The residue was purified by column chromatography on silica gel using PE and EA (4:1) as eluent to give 320 mg of yellow solid. Yield: 73.2 %. Melting point: 96 ^oC -97 ^oC. ^1H NMR (400 MHz, DMSO) δ 7.99 (s, 1H), 7.95 (d, J = 8.0 Hz, 1H), 7.89 (d, J = 8.2 Hz, 1H), 7.63 (t, J = 7.6 Hz, 1H), 7.54 (s, 2H), 7.42 (t, J = 7.7 Hz, 1H).

Compounds 1b and 1c were synthesized similarly as 1a.

1-(2-nitrophenyl)ethylidene-hydrazine (1b) Yellow oil. Yield: 80.2 %. ^{1}H NMR (400 MHz, DMSO) δ 7.76 (d, J = 8.0 Hz, 1H), 7.64 (t, J = 7.6 Hz, 1H), 7.56 (d, J = 7.6 Hz, 1H), 7.47 (t, J = 7.7 Hz, 1H), 6.57 (s, 2H), 1.99 (s, 3H).

4,5-dimethoxy-2-nitrobenzylidene-hydrazine (1c) Yellow solid. Yield: 70.6 %. Melting point: 181 °C -183 °C. ^{1}H NMR (400 MHz, DMSO) δ 8.20 (s, 1H), 7.52 (s, 1H), 7.44 (d, J = 6.1 Hz, 3H), 3.86 (d, J = 10.7 Hz, 6H).

2-nitrobenzyl 2-(6-(cyclobutylamino)benzo[d]thiazol-2-yl)-4,5-dihydrothiazole-4-carboxylate (Cluc-1).

In a 25 mL 2-necked flask fitted with calcium chloride tube, 2-nitrobenzylidene-hydrazine (128 mg; 0.78 mmol) are dissolved in dry methylene chloride (8 mL) to yield a clear, yellow solution. After solution is cooled to -15 °C activated manganese (IV)-oxide (1.68 g; 19 mmol) is added in portions. After completion of the reaction the manganese oxide is removed by filtration (D4 glass frit) and the washed with 5 mL of dry methylene chloride. The clear red solution is transferred to a 50 ml dropping funnel.

The dropping funnel is mounted on a 25 mL 2-necked flask equipped with drying tube and containing a suspension of N-cyclobutylaminoluciferin (90 mg; 0.35 equiv; 0.27 mmol) in dry methanol (5 mL). The suspension is cooled to -3 °C and stirred at 500 rpm then the red solution is added at a speed of 10 drops per minute. The temperature is held constant during the time of the addition which yields a light yellow solution. Then, clear solution is concentrated on a rotary evaporator to yield thick slurry. The residue was purified by column chromatography on silica gel using PE and EA (2:1) as eluent to give 33 mg of yellow solid. Yield: 26.2 %. Melting point: 147 °C -149 °C. ^{1}H NMR (400 MHz, DMSO) δ 8.16 (d, J = 8.0 Hz, 1H), 7.81 (d, J = 8.0 Hz, 3H), 7.66 (d, J = 7.2 Hz, 1H), 7.01 (s, 1H), 6.87 (d, J = 8.7 Hz, 1H), 6.74 (d, J = 6.3 Hz, 1H), 5.59 (dd, J = 19.9, 11.6 Hz, 3H), 3.91 (d, J = 7.3 Hz, 1H), 3.86 – 3.77 (m, 1H), 3.75 – 3.65 (m, 1H), 2.41 (s, 2H), 1.87 (s, 2H), 1.76 (s, 2H). ^{13}C NMR (101 MHz, DMSO) δ170.03, 165.75, 153.39, 148.27, 147.72, 144.63, 138.85, 134.70, 131.69, 129.90, 129.68, 125.45, 124.95, 116.21, 100.81, 78.04, 63.86, 48.23, 34.84, 30.56, 30.52, 15.43. MS (ESI): m/z calcd for Chemical Formula: $C22H21N4O4S2^+$ 469.0999, found 469.0999(M+H)$^+$.

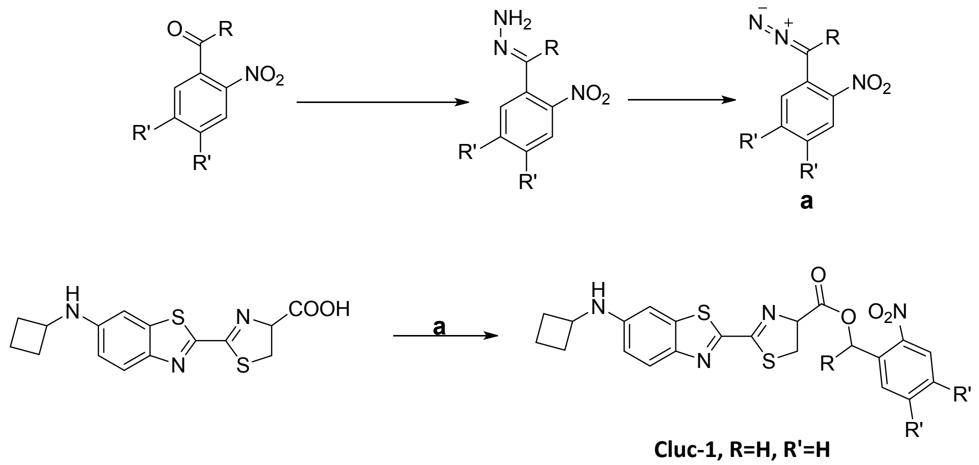

Cluc-1, R=H, R'=H
Cluc-2, R=Me, R'=H
Cluc-3, R=H, R'=OMe

Scheme S1. Synthesis of caged luciferin derivatives.

Compounds Cluc-2 and Cluc-3 were synthesized similarly as Cluc-1 from 1b and 1c.

1-(2-nitrophenyl)ethyl-2-(6-(cyclobutylamino)benzo[d]thiazol-2-yl)-4,5-dihydrothiazole-4-carboxylate (Cluc-2). Yield: 50.1%. ^{1}H NMR (400 MHz, DMSO) δ 8.01 (d, J = 8.1 Hz, 1H), 7.86 – 7.70 (m, 3H), 7.60 (dt, J

= 13.3, 5.0 Hz, 1H), 7.00 (s, 1H), 6.86 (dd, J = 9.0, 1.8 Hz, 1H), 6.74 (d, J = 6.4 Hz, 1H), 6.29 – 6.15 (m, 1H), 5.60 – 5.45 (m, 1H), 3.98 – 3.83 (m, 1H), 3.76 (dd, J = 21.0, 10.1 Hz, 1H), 3.61 (ddd, J = 45.2, 11.2, 7.8 Hz, 1H), 2.45 – 2.31 (m, 2H), 1.85 (dt, J = 17.7, 8.9 Hz, 2H), 1.80 – 1.69 (m, 2H), 1.65 (dd, J = 6.4, 1.7 Hz, 3H). ^{13}C NMR (101 MHz, DMSO) δ 169.61, 165.79, 153.37, 148.26, 147.99, 147.86, 144.62, 138.83, 136.94, 129.74, 127.86, 124.94, 124.80, 116.19, 100.81, 77.90, 69.21, 48.23, 34.88, 30.54, 26.81, 21.88, 15.43. MS (ESI): m/z calcd for Chemical Formula: $C23H23N4O4S2^+$ 483.1155, found 483.1157 $(M+H)^+$.

4,5-dimethoxy-2-nitrobenzyl-2-(6-(cyclobutylamino)benzo[d]thiazol-2-yl)-4,5-dihydrothiazole-4-carboxylate (Cluc-3). ^1H NMR (400 MHz, DMSO) δ 7.79 (t, J = 9.8 Hz, 1H), 7.73 (s, 1H), 7.23 (s, 1H), 7.00 (s, 1H), 6.87 (d, J = 8.9 Hz, 1H), 6.73 (d, J = 6.3 Hz, 1H), 5.60 (dd, J = 15.9, 7.0 Hz, 1H), 5.54 (t, J = 9.9 Hz, 2H), 3.93 (dd, J = 14.9, 7.6 Hz, 1H), 3.87 (d, J = 10.8 Hz, 6H), 3.85 – 3.78 (m, 1H), 3.70 (dd, J = 11.1, 8.3 Hz, 1H), 2.46 – 2.34 (m, 2H), 1.85 (t, J = 14.9 Hz, 2H), 1.81 – 1.67 (m, 2H). ^{13}C NMR (101 MHz, DMSO) δ 170.03, 165.84, 153.75, 153.30, 148.46, 148.29, 144.60, 140.01, 138.80, 126.39, 124.94, 116.22, 111.43, 108.74, 100.79, 78.15, 64.12, 56.73, 56.59, 48.24, 34.97, 30.56, 30.51, 15.42. MS (ESI): m/z calcd for Chemical Formula: $C24H25N4O6S2^+$ 529.1210, found 529.1207 $(M+H)^+$.

Bioluminescence measurements with luciferase.

These caged luciferins were solubilized in DMSO （1.6 mM）and then diluted them at various concentrations (0 μM 1.25 μM, 2.5 μM, 5 μM, 10 μM, 20 μM, 40 μM) in Tris-HCl buffer. Then 100 μL of these compounds (0-40 μM) were assayed in 96-well plates. Finally the equal volume of Tris-HCl buffer containing 20 μg/mL luciferase and 2 mM ATP was added and bioluminescent signals were detected with an exposure time of 1 s.

Bioluminescence imaging in cell.

The ES-2-Fluc cells were cultured in 1640 supplemented with 10% fetal bovine serum (FBS) at 37 ^oC. ES-2-Fluc cells were passed and plated (4×10^4 cells per well) in 96-well plates and cultured 24 h. Then the medium were removed and increasing concentrations of caged luciferin derivatives (1.25μM, 2.5 μM, 5 μM, 10μM, 20 μM, 40 μM) were added. Then the bioluminescence was measured at once with an exposure of 10 s and the bioluminescence intensity was collected. Similarly, ES-2-Fluc at various (0, 1250, 2500, 5000, 10 000, 20 000, and 40 000 cells per well) were passed and plated in 96-well plates and incubated 24 h. Then the caged luciferin derivatives (40 μM) were added after removing the medium. The bioluminescence was measured at once with an exposure of 10 s, and the bioluminescence intensity was collected.

Cytotoxicity test

Therefore, we used the way of Cell Counting Kit-8 to investigate the cell viability. The ES-2-Fluc cells (8×10^3 cells per well) were incubated in 96-well plates and the culture medium was removed after 24 h. Then a variety of dilutions of the luciferin esters (0 μM, 6.25μM, 12.5 μM, 25 μM, 50μM, 100 μM, 200 μM, 400 μM) in complete growth medium were added. Incubating 12 h later, the solution of CCK-8 was added. Finally, the absorbance signals were recorded by a microplate reader at 450 nm.

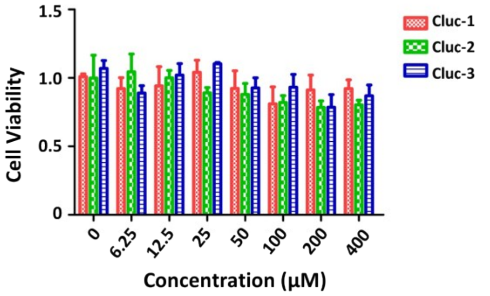

Fig. S1 Cell viability of compound Cluc-1, Cluc-2 and Cluc-3.

Real-time imaging in living cells

The ES-2-Fluc cells were cultured in 1640 supplemented with 10% fetal bovine serum (FBS) at 37 °C. ES-2-Fluc cells were passed and plated (4 × 10^4 cells per well) in 96-well plates and cultured 24 h. Then the medium were removed and increasing concentrations of caged luciferin derivatives (40 μM) were added. Luminescence signals were immediately recorded every 5 min with an exposure time of 10 s.

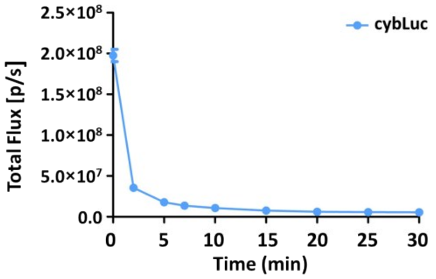

Fig. S2 The rate of the bioluminescence reaction for cybLuc evaluated as the change of the total light intensity in the cell over time.

In vivo Bioluminescent Imaging

Pathogen-free luciferase-expressing transgenic mice which were about 24 g of male were provided. After being anesthetized with chloral hydrate(8%), the N-cyclobutylaminoluciferin and caged luciferin derivatives were injected intraperitoneally（100 μL of 1 mM solutions in NS）into mice. Then bioluminescence imaging was immediately acquired with an exposure time of 1 s and the light signal was recorded every ten min until it disappeared. Each compound was evaluated using at least three mice.

Inhibitory activity

These caged luciferins were solubilized in DMSO and then diluted them at various concentrations (0 μM 5 μM, 10 μM, 20 μM, 40 μM, 80 μM, 160 μM) in Tris-HCl buffer. Then 100 μL of these caged luciferins (0-160 μM) and 50 μL of cybLuc (2 μM) were assayed in 96-well plates. Finally 50 μL of Tris-HCl buffer containing 20 μg/mL luciferase and 2 mM ATP was added and bioluminescent signals were detected with an exposure time of 1 s.

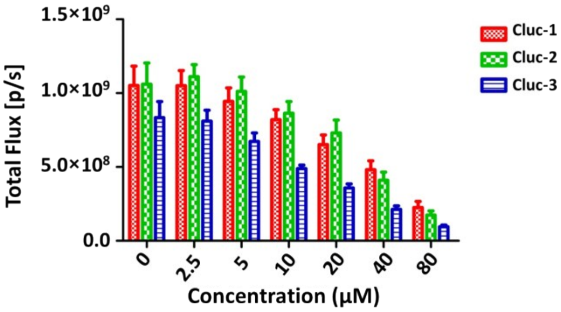

Fig. S3 Concentration–response for Clucs in a recombinant firefly luciferase inhibition assay

^{1}H NMR, ^{13}C NMR and ESI-HRMS for compounds

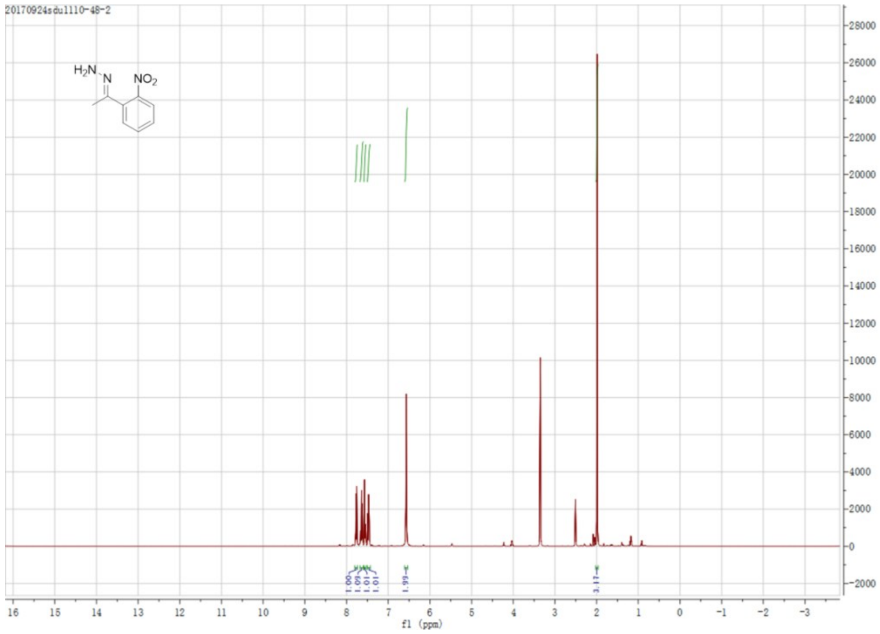

^{1}H NMR spectrum of 1b

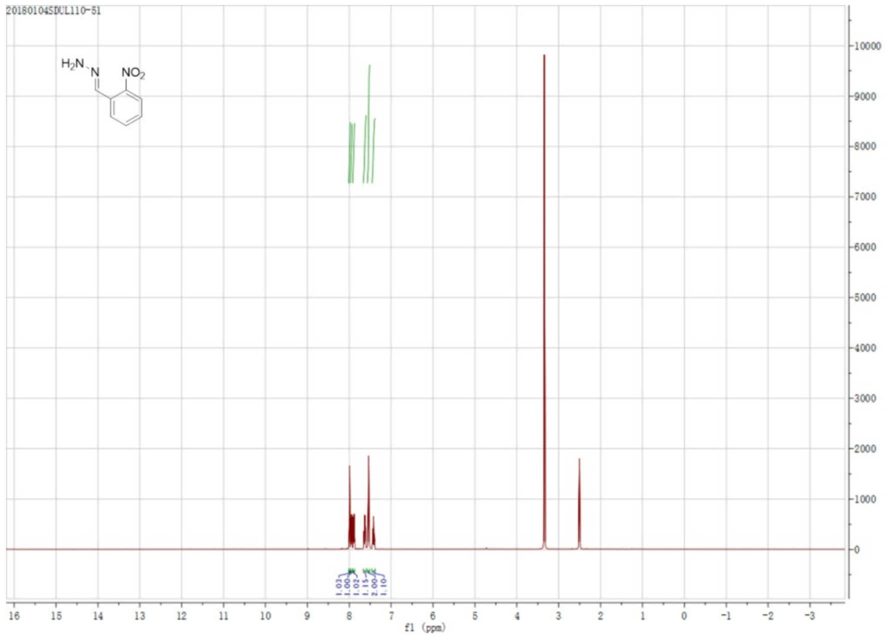

^{1}H NMR spectrum of 1a

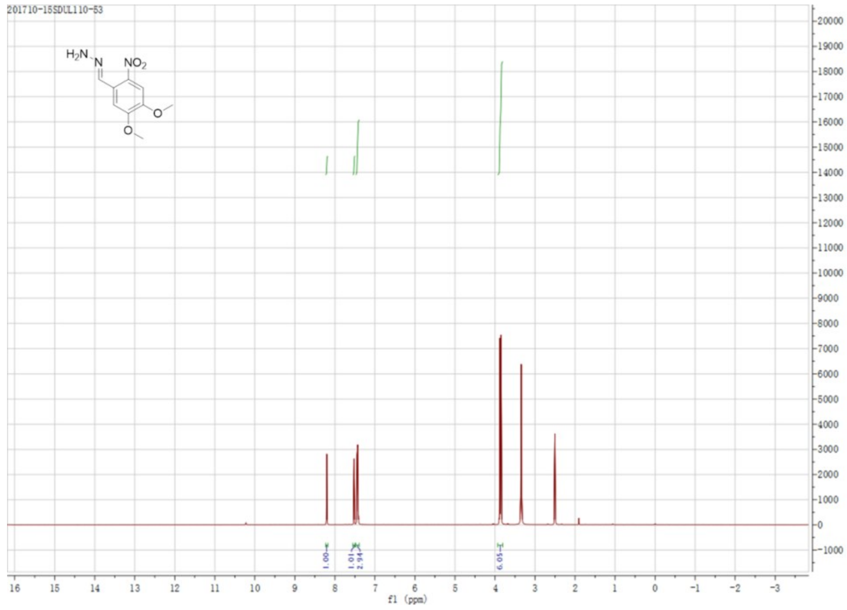

^{1}H NMR spectrum of 1c

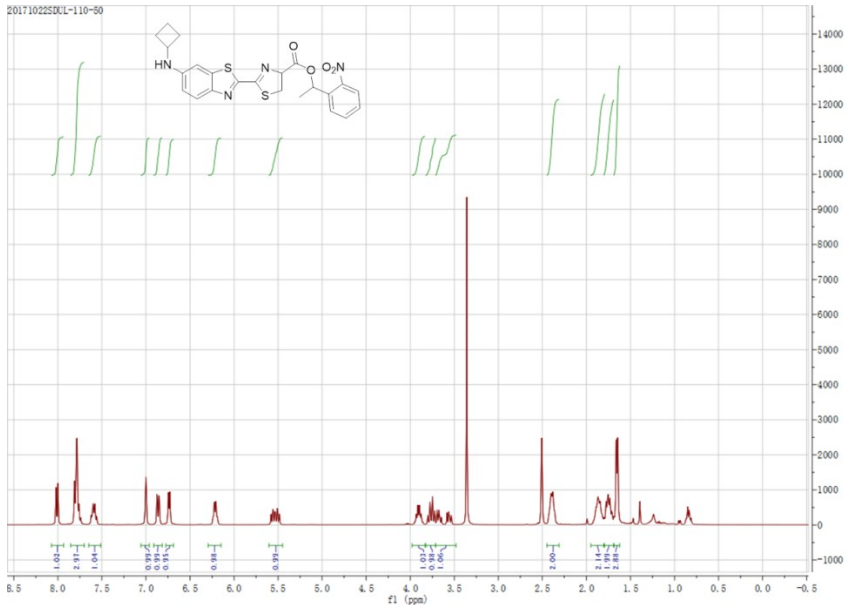

^{1}H NMR spectrum of Cluc-2

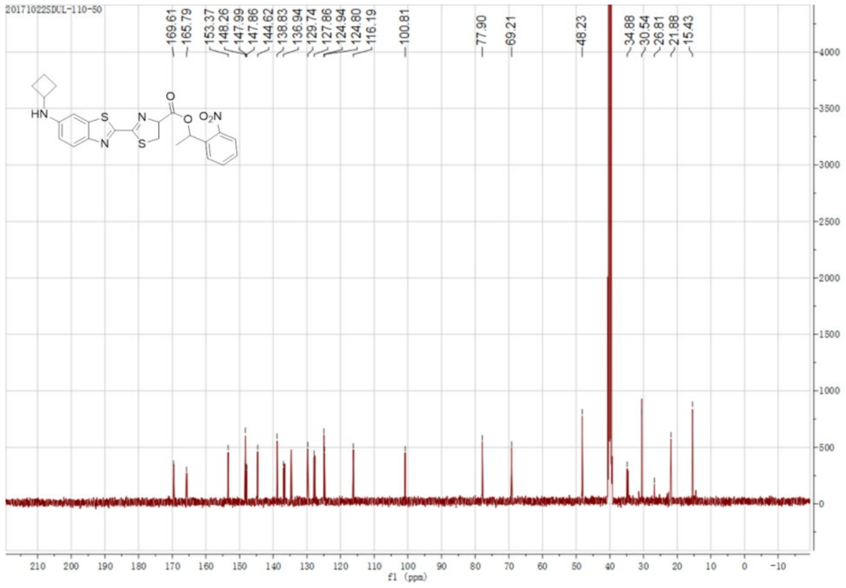

^{13}C NMR spectrum of Cluc-2

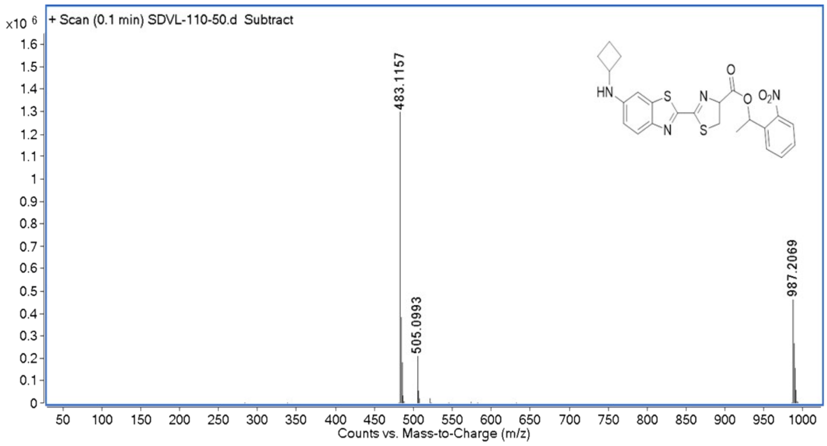

HRMS spectrum of Cluc-2

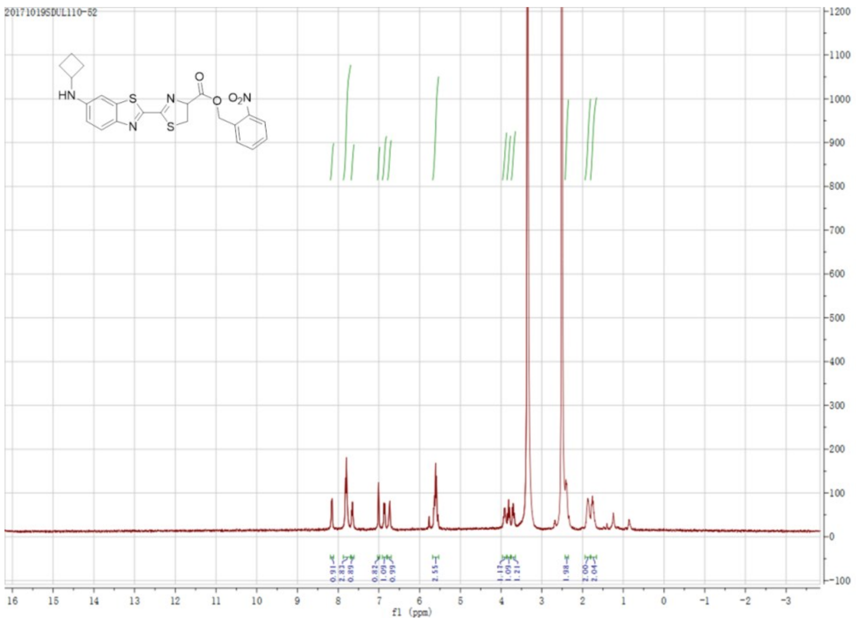

^1H NMR spectrum of Cluc-1

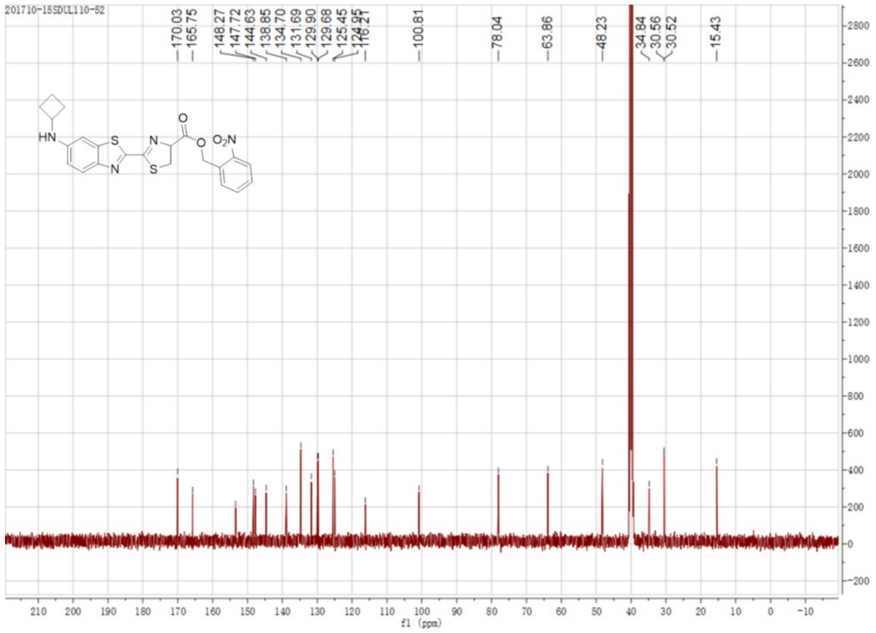

^{13}C NMR spectrum of Cluc-1

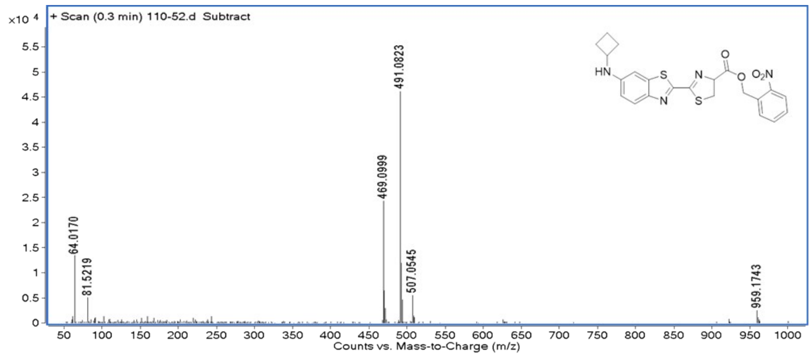

HRMS spectrum of Cluc-1

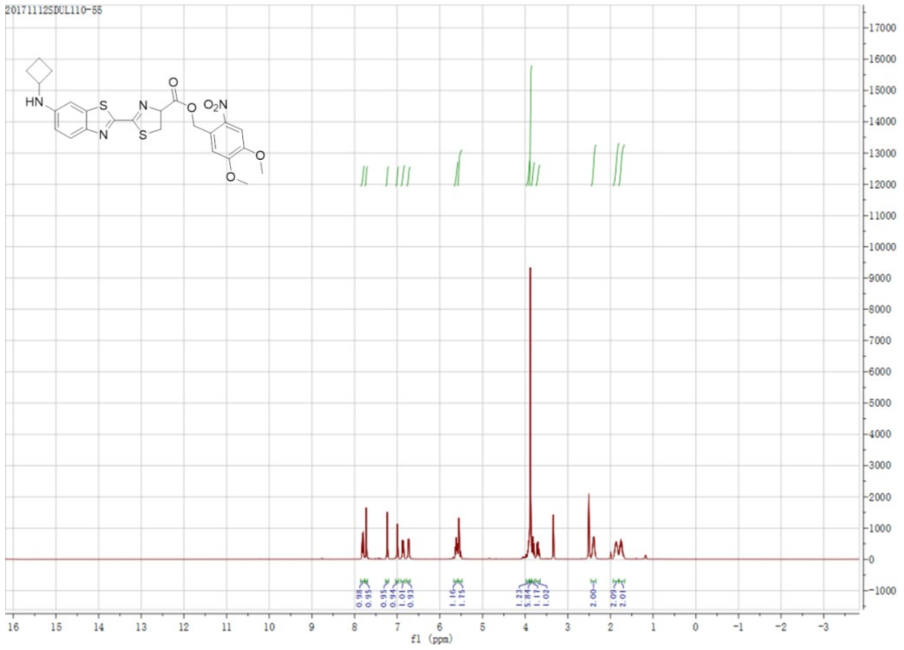

^{1}H NMR spectrum of Cluc-3

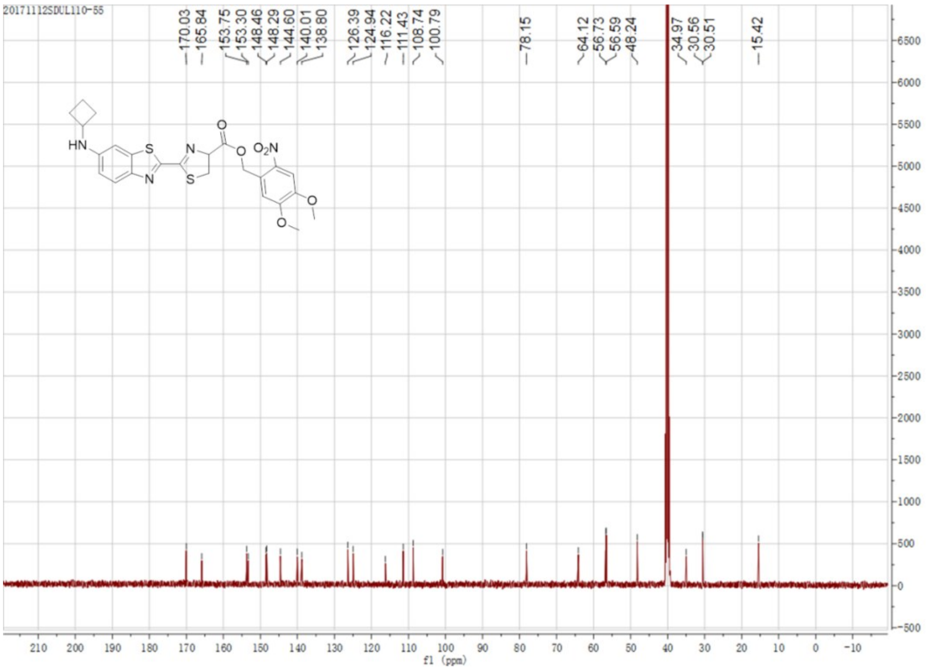

^{13}C NMR spectrum of Cluc-3

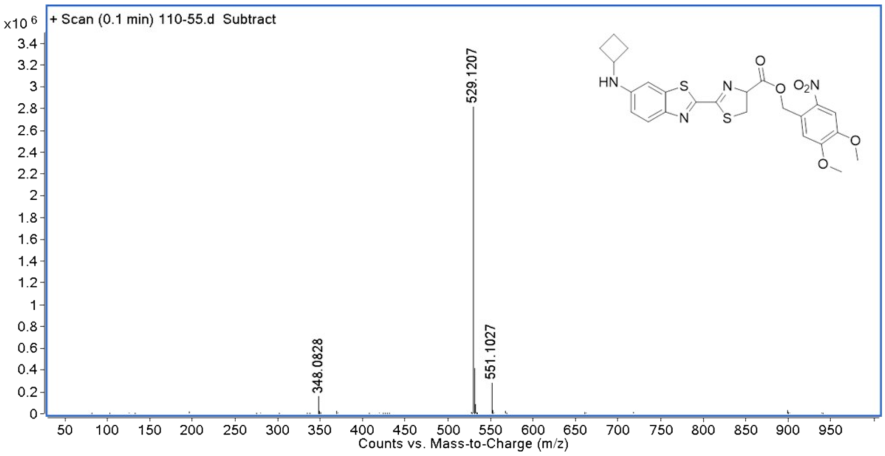

HRMS spectrum of Cluc-3
